# Moderate-intensity versus high-intensity statin therapy in Korean patients with angina undergoing percutaneous coronary intervention with drug-eluting stents: A propensity-score matching analysis

**DOI:** 10.1371/journal.pone.0207889

**Published:** 2018-12-07

**Authors:** Mahn-Won Park, Gyung-Min Park, Seungbong Han, Yujin Yang, Yong-Giun Kim, Jae-Hyung Roh, Hyun Woo Park, Jon Suh, Young-Rak Cho, Ki-Bum Won, Soe Hee Ann, Shin-Jae Kim, Dae-Won Kim, Sung Ho Her, Sang-Gon Lee

**Affiliations:** 1 Department of Cardiology, Daejeon St. Mary's Hospital, The Catholic University of Korea, Daejeon, Korea; 2 Department of Cardiology, Ulsan University Hospital, University of Ulsan College of Medicine, Ulsan, Korea; 3 Department of Applied Statistics, Gachon University, Seongnam, Korea; 4 Department of Cardiology, Chungnam National University Hospital, Chungnam National University School of Medicine, Daejeon, Korea; 5 Department of Cardiology, Soon Chun Hyang University Hospital Bucheon, Bucheon, Korea; 6 Department of Cardiology, Dong-A University Hospital, Busan, Republic of Korea; Bern University Hospital, SWITZERLAND

## Abstract

**Objectives:**

It is unclear whether high-intensity statin therapy provides incremental clinical benefits over moderate-intensity statin therapy in Asian patients with angina. This study sought to compare the clinical outcomes of moderate- and high-intensity statin therapies in patients undergoing percutaneous coronary intervention (PCI) for angina in Korean patients.

**Methods:**

Based on the national health insurance claims data in South Korea, patients aged 18 years or older without a known history of coronary artery disease, who underwent PCI with drug-eluting stents due to angina between 2011 and 2015, were enrolled. According to the intensity of statin therapy, patients were categorized into moderate-intensity statin therapy (n = 23,863) and high-intensity statin therapy (n = 9,073) groups. The primary endpoint, defined as a composite of all-cause death and myocardial infarction, was compared between the two groups using a propensity-score matching analysis.

**Results:**

During the follow-up period (median, 2.0 years; interquartile range, 1.1–3.1), 1,572 patients had 1,367 deaths and 242 myocardial infarctions. After propensity-score matching, there were 8,939 matched pairs. There was no significant difference in the incidence of the primary endpoint between the two groups (adjusted hazard ratio of high-intensity statin therapy, 1.093; 95% confidence interval: 0.950–1.259; p = 0.212).

**Conclusions:**

In Korean patients undergoing PCI with drug-eluting stents for angina, the high-intensity statin therapy did not provide additional clinical benefits over the moderate-intensity statin therapy.

## Introduction

Low-density lipoprotein (LDL) cholesterol lowering therapy with statins improved clinical outcomes in patients with and without coronary artery disease (CAD) in previous randomized trials [1–4]. The current guidelines recommend the use of statins, especially high-intensity statin therapy, in patients with established CAD [5, 6]. However, this has not yet been properly incorporated into clinical practice [7]. On the other hand, East Asian populations may have better statin responsiveness, lower baseline LDL cholesterol, and greater vulnerability to the side effects of statin therapy compared to Western populations [8, 9]. In addition, a recent randomized study failed to demonstrate the incremental clinical efficacy of high-intensity statin therapy in an East Asian population [10]. Therefore, using claims data of the National Health Insurance (NHI) in South Korea, we sought to 1) evaluate the clinical impact of statin therapy at hospital discharge on the prognosis, and 2) compare the clinical outcomes of moderate- versus high-intensity statin therapies in Korean patients undergoing percutaneous coronary intervention (PCI) with drug-eluting stents (DES) for angina.

## Methods

### Data sources

South Korea has a NHI system. Therefore, all healthcare providers had to join this system on a fee-for-service basis. The Health Insurance Review & Assessment Service (HIRA) is a quasi-governmental organization that systematically reviews medical fees to minimize the risk of redundant and unnecessary medical services. Consequently, all NHI claims are reviewed by the HIRA [11]. For this study, data from the January 2011 to June 2016 claims records of the HIRA were used. Diagnosis codes were used according to the International Classification of Diseases, 10th Revision (ICD-10). In addition, specific information about the drugs, devices, and procedures were identified by codes from the HIRA database [11]. This study was approved by the local Institutional Review Board of Ulsan University Hospital, Ulsan, Korea.

### Study population

From the claims database of the HIRA between July 2011 and June 2015, we identified patients aged 18 years and older who had undergone PCI (M6551, M6552, M6561-4, M6571, and M6572) with DES (J5083XXX) for the diagnosis of CAD (ICD-10 codes I20.X-I25.X). Patients with at least 6 months of eligibility prior to the index day were selected. Specifically, patients with an index procedure for the diagnosis of acute myocardial infarction (MI) (I21.X-I22.X) were excluded in order to focus on patients with angina. We excluded patients if the HIRA database indicated that they had a previous history of CAD (ICD-10 codes I20.X–25.X) within 6 months of the index day to ensure that it was the patients’ first episode of angina. Additionally, patients who died during hospitalization after the index procedure were excluded to create a more homogeneous population by reducing patient-related confounding factors.

### Study variables

The ICD-10 codes were used to identify comorbid conditions such as diabetes, diabetes with chronic complications, hyperlipidemia, hypertension, congestive heart failure, arrhythmia, valvular disease, peripheral vascular disease, cerebrovascular disease, chronic pulmonary disease, moderate to severe liver disease, renal disease, cancer, and rheumatic disease [12, 13]. The Charlson comorbidity index was obtained from the ICD-10 codes [12].

In the HIRA database, all prescribed medications were exclusively recorded with rigorous accuracy. In the present study, we identified the medications used, such as anti-platelet agents, statins, beta-blockers, and angiotensin-converting enzyme inhibitors/angiotensin receptor blockers [14]. At discharge, patients were categorized into low-intensity (simvastatin 10 mg, pravastatin 5–20 mg, lovastatin 20 mg, fluvastatin 20–40 mg, pitavastatin 1 mg), moderate-intensity (atorvastatin 10–20 mg, rosuvastatin 5–10 mg, simvastatin 20–80 mg, pravastatin 40–80 mg, lovastatin 40 mg, fluvastatin XL 80 mg, pitavastatin 2–4 mg), and high-intensity (atorvastatin 40–80 mg, rosuvastatin 20–40 mg) statin therapy groups according to the 2013 ACC/AHA guidelines [15]. Moreover, to compare clinical outcomes according to the intensity of statin therapy in patients undergoing PCI for stable CAD, we excluded patients taking ezetimibe containing statins or ≥ 2 statins.

### Clinical outcomes

The primary endpoint of this study was a composite of all-cause death and MI. In patients with multiple primary events, the first event was considered to be the component of the composite outcome. Death was identified by all in- and out-patient claims that indicated death. MI was defined using the hospital discharge databases of the HIRA (ICD-10 codes I21.X–22.X) [16]. In this current study, for the evaluation of clinical outcomes, the HIRA database was used until June 2016.

### Statistical analysis

All baseline patient characteristic and comorbid conditions were summarized as mean ± standard deviation or frequency (percentage) for continuous or categorical variables, respectively. Categorical data were compared using Chi-square or Fisher’s exact tests. Continuous variables with normal distributions were compared using the Student’s t-test, and those without normal distributions were compared using the Mann–Whitney U test. Cumulative incidence rates for clinical outcomes between the statin and non-statin therapy groups or between the high- and moderate- intensity statin therapy groups were estimated using the Kaplan–Meier method. We compared the cumulative incidences between the two groups using the log-rank test. Significance was defined as p<0.05 for all two-tailed tests. We used the propensity-score matching method to reduce potential confounding factors in the demographics and comorbid conditions between the two comparison groups. The propensity-scores were derived nonparametrically using the variables of age, gender, comorbid conditions, the number of stents, the medications at discharge, and the Charlson comorbidity index. Nonparametric propensity-score estimation was useful because there was no need to fit the fully corrected parametric model. Propensity-score matching was performed by nearest-neighbor matching using a caliper size of 0.2 multiplied by the standard deviation for linearly transformed propensity scores (logit-transformation). The balance of covariates was measured by their standardized differences in means. All the standardized differences for the baseline variables were less than 0.05 (5%), so that all pretreatment variables were balanced (S1 and S2 Figs). Furthermore, we conducted the paired t-test or the McNemar test for continuous or categorical variables to check out the covariate balance between the two matched groups. In the propensity-score matched cohort, the risks of clinical events were compared via the Cox regression model with robust standard errors accounting for the clustering of matched pairs. Data analysis was performed using the R software version 3.3.1 (R Foundation for Statistical Computing, Vienna, Austria; www.r-project.org).

## Results

### Study population and characteristics

**Fig 1:**
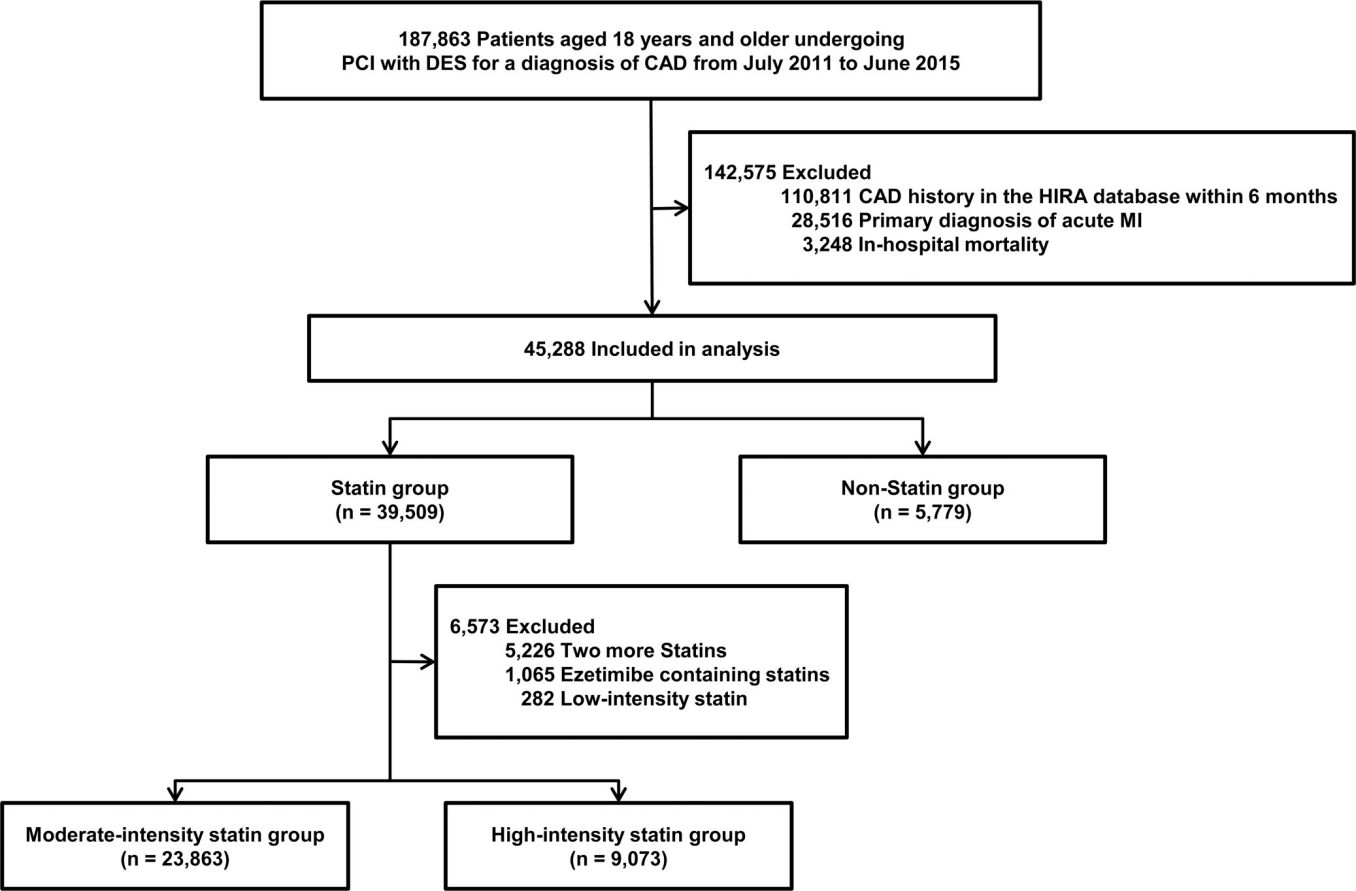
Overview of the study population. CAD = coronary artery disease; DES = drug-eluting stents; HIRA = the Health Insurance Review & Assessment Service; MI = myocardial infarction; PCI = percutaneous coronary intervention.

Between July 2011 and June 2015, a total of 187,863 patients aged 18 years and older undergoing PCI with DES for CAD were identified from the claims database of HIRA. Among them, 45,288 patients met the eligibility criteria and were selected as the study population (Fig 1). Table 1 lists the baseline characteristics of study participants. The mean age of the study participants was 64.9 ± 11.4 years and 30,171 (66.6%) were men. Diabetes mellitus, hyperlipidemia, and hypertension were observed in 14,557 (32.1%), 19,790 (43.7%), and 26,619 (58.8%) patients, respectively. At discharge, anti-platelet agents, statins, beta-blockers, and angiotensin converting enzyme inhibitors or angiotensin receptor blockers were provided to 44,980 (99.3%), 39,509 (87.2%), 29,139 (64.3%), and 28,517 (63.0%) patients, respectively. In addition, specific anti-platelet agents were presented in S1 Table.

**Table 1:** Characteristics of patients undergoing percutaneous coronary intervention with drug-eluting stents for angina according to statin therapy.

	Overall (n = 45,288)					Overall (n = 32,936)		
Characteristics	Statin (n = 39,509)	Non-statin (n = 5,779)			P Value	Moderate-intensity statin (n = 23,863)	High-intensity statin (n = 9,073)	P Value
Age, years	64.7±11.5	66.6±10.9			<0.001	65.3±11.3	63.4±11.7	<0.001
Gender male, no. (%)	26,358 (66.7%)	3,813 (66.0%)			0.269	15,638 (65.5%)	6,337 (69.8%)	<0.001
Enrolled number, no. (%)					<0.001			<0.001
July 2009 to June 2010	8,308 (21.0%)	1,858 (32.2%)				5,665 (23.7%)	1,354 (14.9%)	
July 2010 to June 2011	9,289 (23.5%)	1,619 (28.0%)				5,960 (25.0%)	1,714 (18.9%)	
July 2011 to June 2012	10,695 (27.1%)	1,280 (22.1%)				6,363 (26.7%)	2,510 (27.7%)	
July 2012 to June 2013	11,217 (28.4%)	1,022 (17.7%)				5,875 (24.6%)	3,495 (38.5%)	
Comorbid conditions, no. (%)								
Diabetes	12,253 (31.0%)	2,167 (37.5%)			<0.001	7,724 (32.4%)	2,538 (28.0%)	<0.001
Diabetes with chronic complications	117 (0.3%)	20 (0.3%)			0.521	65 (0.3%)	31 (0.3%)	0.304
Hyperlipidemia	17,106 (43.3%)	2,684 (46.4%)			<0.001	10,798 (45.2%)	3,528 (38.9%)	<0.001
Hypertension	22,811 (57.7%)	3,808 (65.9%)			<0.001	14,364 (60.2%)	4,843 (53.4%)	<0.001
Congestive heart failure	2,640 (6.7%)	564 (9.8%)			<0.001	1,728 (7.2%)	484 (5.3%)	<0.001
Arrhythmia	3,143 (8.0%)	665 (11.5%)			<0.001	2,038 (8.5%)	648 (7.1%)	<0.001
Valvular disease	169 (0.4%)	48 (0.8%)			<0.001	117 (0.5%)	21 (0.2%)	0.001
Peripheral vascular disease	4,505 (11.4%)	811 (14.0%)			<0.001	2,921 (12.2%)	904 (10.0%)	<0.001
Cerebrovascular disease	5,390 (13.6%)	1,060 (18.3%)			<0.001	3,366 (14.1%)	1,132 (12.5%)	<0.001
Chronic pulmonary disease	6,530 (16.5%)	1,043 (18.0%)			0.004	4,075 (17.1%)	1,403 (15.5%)	<0.001
Moderate to severe liver disease	20 (0.1%)	5 (0.1%)			0.240	16 (0.1%)	3 (0.03%)	0.313
Renal disease	1,872 (4.7%)	508 (8.8%)			<0.001	1,220 (5.1%)	321 (3.5%)	<0.001
Cancer	1,035 (2.6)	227 (3.9)			<0.001	654 (2.7)	224 (2.5)	0.180
Rheumatic disease	79 (0.2%)	15 (0.3%)			0.352	49 (0.2%)	20 (0.2%)	0.788
Charlson comorbidity index	1.32±1.37	1.63±1.51			<0.001	1.38±1.40	1.17±1.28	<0.001
Number of drug-eluting stents	1.42±0.67	1.34±0.61			<0.001	1.41±0.65	1.45±0.69	<0.001
Medications at discharge, no. (%)								
Anti-platelet agent	39,437 (99.8%)	5,543 (95.9%)			<0.001	23,821 (99.8%)	9,055 (99.8%)	0.666
Beta-blocker	26,250 (66.4%)	2,889 (50.0%)			<0.001	15,121 (63.4%)	6,511 (71.8%)	<0.001
ACEI/ARB	25,760 (65.2%)	2,757 (47.7%)			<0.001	15,345 (64.3%)	5,837 (64.3%)	0.969

Data are expressed as n (%) and mean ± SD. ACEI = angiotensin converting enzyme inhibitor; ARB = angiotensin receptor blocker

### Non-statin versus statin therapy

**Fig 2:**
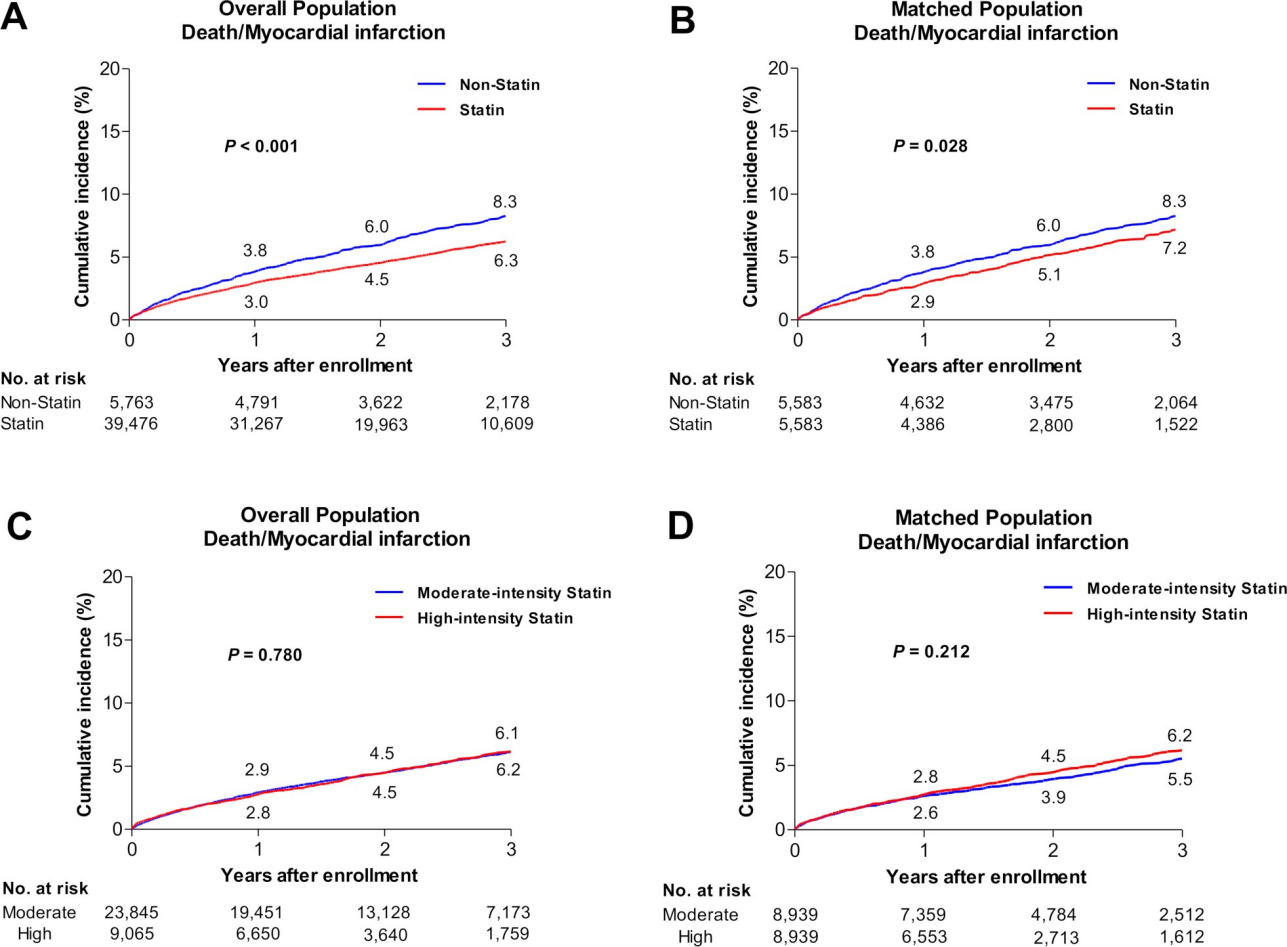
Cumulative incidence rates for clinical outcomes in the study population. Cumulative incidence rates for all-cause death/myocardial infarction in overall (A) and matched (B) population between the statin and non-statin therapy groups; all-cause death/myocardial infarction in overall (C) and matched (D) population between the high- and moderate-intensity statin therapy groups. The numbers in each figure represent the cumulative incidence rates at each time points.

According to the discharge medications, patients were categorized into non-statin (n = 5,779) and statin (n = 39,509) therapy groups (Fig 1). Patients with non-statin therapy were older and had more comorbid conditions than those with statin therapy (Table 1). During the follow-up period (median, 2.1 years; interquartile range, 1.2–3.2), 2,328 patients had 2,041 deaths and 347 MIs (S2 Table). After propensity-score matching, there were 5,583 matched pairs. In the matched cohort, there were no other significant differences for any of the covariates between the two groups (Table 2). The adjusted incidence of the primary endpoint defined as a composite of death and MI was significantly lower in the statin therapy group (adjusted hazard ratio [aHR] of statin therapy, 0.844; 95% confidence interval [CI]: 0.726–0.982; p = 0.028) (Table 3). Fig 2 shows the cumulative incidence rates for clinical outcomes between the two groups.

**Table 2:** Characteristics of propensity-score matched patients according to statin therapy.

	Overall (n = 11,166)					Overall (n = 17,878)		
Characteristics	Statin (n = 5,583)	Non-statin (n = 5,583)			P Value	Moderate-intensity statin (n = 8,939)	High-intensity statin (n = 8,939)	P Value
Age, years	66.4±11.2	66.5±10.9			0.298	63.8±11.5	63.6±11.6	0.763
Gender male, no. (%)	3,641 (65.2%)	3,668 (65.7%)			0.842	6,137 (68.7%)	6,211 (69.5%)	0.118
Comorbid conditions, no. (%)								
Diabetes	1,988 (35.6%)	2,074 (37.1%)			0.025	2,548 (28.5%)	2,528 (28.3%)	0.959
Diabetes with chronic complications	20 (0.4%)	20 (0.4%)			0.429	28 (0.3%)	30 (0.3%)	0.896
Hyperlipidemia	2,498 (44.7%)	2,543 (45.5%)			0.237	3,509 (39.3%)	3,520 (39.4%)	0.742
Hypertension	3,562 (63.8%)	3,661 (65.6%)			0.508	4,840 (54.1%)	4,830 (54.0%)	0.910
Congestive heart failure	509 (9.1%)	540 (9.7%)			0.645	486 (5.4%)	484 (5.4%)	0.516
Arrhythmia	604 (10.8%)	632 (11.3%)			0.737	677 (7.6%)	648 (7.2%)	0.863
Valvular disease	54 (1.0%)	47 (0.8%)			0.232	23 (0.3%)	21 (0.2%)	0.626
Peripheral vascular disease	742 (13.3%)	778 (13.9%)			0.636	904 (10.1%)	902 (10.1%)	0.437
Cerebrovascular disease	996 (17.8%)	975 (17.5%)			0.146	1,136 (12.7%)	1,128 (12.6%)	0.073
Chronic pulmonary disease	1,011 (18.1%)	1,013 (18.1%)			0.903	1,420 (15.9%)	1,395 (15.6%)	0.536
Moderate to severe liver disease	4 (0.1%)	5 (0.1%)			0.999	1 (0.01%)	3 (0.03%)	0.617
Renal disease	470 (8.4%)	488 (8.7%)			0.150	326 (3.6%)	321 (3.6%)	0.408
Cancer	202 (3.6)	218 (3.9)			0.034	229 (2.6)	223 (2.5)	0.476
Rheumatic disease	12 (0.2%)	15 (0.3%)			0.999	21 (0.2%)	19 (0.2%)	0.635
Charlson comorbidity index	1.59±1.51	1.62±1.50			0.099	1.20±1.30	1.18±1.28	0.964
Number of drug-eluting stents	1.33±0.60	1.33±0.60			0.992	1.44±0.68	1.45±0.69	0.714
Medications at discharge, no. (%)								
Anti-platelet agent	5,511 (98.7%)	5,517 (98.8%)			0.099	8,919 (99.8%)	8,921 (99.8%)	0.999
Beta-blocker	3,025 (54.2%)	2,872 (51.4%)			0.894	6,431 (71.9%)	6,378 (71.4%)	0.339
ACEI/ARB	2,832 (50.7%)	2,743 (49.1%)			0.981	5,711 (63.9%)	5,751 (64.3%)	0.790

Data are expressed as n (%) and mean ± SD. ACEI = angiotensin converting enzyme inhibitor; ARB = angiotensin receptor blocker

**Table 3:** Clinical outcomes in patients undergoing percutaneous coronary intervention with drug-eluting stents for angina according to statin therapy.

Propensity-score matching analysis	Statin compared with Non-statin		
	Hazard ratio (95% CI)	P value	
All-cause death/myocardial infarction	0.844 (0.726–0.982)	0.028	
Propensity-score matching analysis	High-intensity statin compared with Moderate-intensity statin		
	Hazard ratio (95% CI)	P value	
All-cause death/myocardial infarction	1.093 (0.950–1.259)	0.212	

CI = confidence interval.

### Moderate-intensity statin versus high-intensity statin therapy

We also analyzed clinical outcomes according to the intensity of statin therapy. Patients were classified into moderate-intensity (n = 23,863) and high-intensity (n = 9,073) statin therapy groups (Fig 1). S3 Table shows frequency and doses of statins in moderate- and high-intensity statin groups. Patients receiving moderate-intensity therapy were older and had more comorbid conditions than those receiving high-intensity statin therapy (Table 1). Baseline characteristics of patients taking ezetimibe containing statins or ≥2 statins are presented in S4 Table. During the follow-up period (median, 2.0 years; interquartile range, 1.1–3.1), 1,572 patients had 1,367 deaths and 242 MIs (S2 Table). After propensity-score matching, there were 8,939 matched pairs. In the matched cohort, there were no other significant differences for any of the covariates between both groups (Table 2). The incidence of the primary endpoint did not differ between the two groups (aHR of high-intensity statin therapy, 1.093; 95% CI: 0.950–1.259; p = 0.212). The cumulative incidence rates for clinical outcomes between the two groups are presented in Fig 2.

## Discussion

In the present analysis using NHI claims data in South Korea, our main findings were as follows: 1) In South Korean patients undergoing PCI with DES for angina, statin therapy at hospital discharge was associated with better clinical outcomes; 2) However, high-intensity statin therapy did not provide additional clinical benefits over moderate-intensity statin therapy after adjusting for potentially confounding variables.

There are few data available regarding whether statin therapy at hospital discharge improves clinical outcomes in patients undergoing PCI with DES for angina. There is still a lack of evidence regarding whether high-intensity statin therapy provides additional clinical benefits in these patients. In several previous studies, although the higher intensity statin therapy showed better clinical outcomes over the lower intensity statin therapy, these studies analyzed limited populations, with particular focusing on Western populations [17, 18]. In addition, owing to relatively low hard clinical event rates in patients with angina, it was difficult to make definitive conclusions as to the incremental clinical benefits of higher intensity statin therapy for hard clinical events, such as death and MI. However, our study has the advantage of well-controlled and reliable data from the nationwide database in Korea (i.e., a quasi-governmental organization) that enabled qualified analyses for the moderate- versus high-intensity statin therapies in angina patients undergoing PCI with DES [11, 16].

Statin therapy with absolute reduction in LDL cholesterol demonstrated consistent prognostic benefits for primary as well as secondary prevention in previous randomized trials [1–4]. In addition, the clinical and experimental data showed the pleiotropic effects of statins such as anti-inflammatory activity, improvement in endothelial function, reduction of oxidative stress, and antithrombotic activity [19]. Based on these unique properties, in patients with CAD, earlier statin therapy provided better clinical outcomes regardless of LDL cholesterol levels or clinical presentations [7, 20–22]. In the present study, consistent with the findings of previous observational and randomized trials [7, 20–22], we reaffirmed that statin therapy at hospital discharge was associated with improved hard clinical outcomes such as all-cause death or MI after coronary revascularization. Therefore, given that our study has contributed further evidence of the consistent clinical benefits of statins for secondary prevention, further implementation of statin therapy should be required in patients with angina undergoing coronary revascularization.

In the present study, after adjustments for possible confounding variables, high-intensity statin therapy did not provide incremental clinical benefits over moderate-intensity statin therapy in patients undergoing PCI with DES for angina. A current accepted guideline has advocated the use of high-intensity statin therapy in patients with established CAD [5]. However, this guideline may not be directly applicable to Asian patients who have different clinical and genetic backgrounds compared to Westerners. A pharmacokinetic study also indicated that the greater effect of statins could be due in part to the difference in statin pharmacokinetics between East Asian and Western patients [23]. In prior studies with East Asian populations, lower-dose statin therapy showed similar therapeutic effects to those observed in Western populations using higher-dose statin therapy [24, 25]. In addition, serial intravascular ultrasound studies with East Asian patients demonstrated that the regression of coronary atherosclerosis could be achieved by moderate-intensity statin therapy [25–27]. However, these findings were only observed in the context of high-intensity statin therapy for Western patients [28]. Furthermore, a randomized trial observed that East Asian patients were more vulnerable to adverse effects rather than Western patients [9]. In accordance with these lines of available evidence, our findings highlight that the therapeutic effects of statins in East Asian populations might differ from those in Western populations, indicating that lower intensity statin therapy could be sufficient to attenuate future cardiovascular risks with minimal adverse effects in these populations.

In another Korean retrospective study with stable coronary artery disease, Lee et al. compared clinical outcomes between patients with statins equivalent to or weaker than atorvastatin 10 mg (group 1, n = 181) and those with statins equivalent to or stronger than atorvastatin 20 mg (group 2, n = 264). During a median follow-up of 4.5 years, major adverse cardiac events defined as the composite of cardiovascular death, non-fatal MI, and coronary revascularization was significantly lower in group 2 (16.6% in group 1 versus 4.5% in group 2, p<0.001). However, there was no significant difference in the incidence of the composite of cardiovascular death and non-fatal MI between the two groups (1.7% in group 1 versus 1.1% in group 2). Most patients (n = 394) received moderate-intensity statin therapy and only a small number of patients (n = 51) took high intensity statins [29]. Moreover, in a recent Japanese randomized trial, moderate-intensity statin therapy (pitavastatin 4 mg/day) significantly reduced cardiovascular events in Japanese patients with stable coronary artery disease compared with low-intensity statin therapy (pitavastatin 1 mg/day) [30]. Therefore, taken together with our study, these findings suggest that moderate-intensity statin therapy may be an initial treatment in patients with stable CAD.

On the other hand, stronger statins could be beneficial in very high-risk East Asian patients such as those with left main disease, multi-vessel disease, or diabetes [31]. In these patients, stronger statins with target LDL cholesterol levels of <70 mg/dL or a reduction of at least 50% if the baseline LDL cholesterol is between 70 and 135 mg/dL would be more appropriate according to the current guidelines [6, 32]. Therefore, even in East Asians patients undergoing coronary revascularization for angina, high-intensity statin therapy may be helpful in very high-risk patients.

Our study had several limitations. First, the present study was based on administrative data from the HIRA in South Korea. Similar to previous studies using administrative databases, our study lacked patient clinical data and test findings. Thus, our findings might be limited by uncertainties in unmeasured confounding variables that may affect the management of patients [13, 33]. In addition, the current analysis did not distinguish between stable angina and unstable angina. Second, although we used the database by the quasi-governmental organization, there was a possibility that these data could not have fully reflected diagnosis and clinical outcomes. Additionally, we did not specify the cause of death. Third, the information about medical therapy was only obtained at discharge. However, a recent observational study showed that the majority of patients did not adjust the intensity of their statin therapy after hospital discharge despite experiencing an acute coronary syndrome [34]. Fourth, in the current study, there is a trend towards more frequent prescription of high-intensity statin therapy in a later period. This may have an effect on clinical outcomes in the high-intensity statin group with shorter follow-up. Therefore, our findings need to be confirmed in other prospective studies with long-term clinical follow-up. Finally, the present study only included a Korean population. Therefore, it might not be possible to generalize our findings to other countries. However, since East Asian patients share similar risk factors and specific characteristics of cardiovascular diseases [11, 35], we believe that our findings have the potential to be applied to other East Asian populations, such as Japanese and Chinese populations.

## Conclusions

In Korean patients undergoing PCI with DES for angina, the efficacy of moderate-intensity statin therapy was comparable to that of high-intensity statin therapy in terms of improved clinical outcomes. Our findings suggest that moderate-intensity statin therapy may be an initial treatment strategy with comparable clinical efficacy compared to high-intensity statin therapy in East Asian patients with angina undergoing PCI. However, these findings should be confirmed in future randomized clinical trials.

## Supporting information

S1 Fig Covariate balance in propensity-score matched patients between statin and non-statin groups. (DOCX)

S2 Fig Covariate balance in propensity-score matched patients between moderate- and high-intensity statin groups. (DOCX)

S1 Table Specific anti-platelet agents in patients undergoing percutaneous coronary intervention with drug-eluting stents for angina according to statin therapy. (DOCX)

S2 Table Clinical outcomes according to statin therapy. (DOCX)

S3 Table Frequency and doses of statins in moderate- and high-intensity statin therapy groups. (DOCX)

S4 Table Characteristics of patients according to statin therapy. (DOCX)

## Abbreviations

CAD: coronary artery disease
CI: confidence interval
DES: drug-eluting stents
HIRA: Health Insurance Review & Assessment Service
HR: hazard ratio
ICD-10: International Classification of Diseases, 10th Revision
LDL: Low-density lipoprotein
MI: myocardial infarction
NHI: National Health Insurance
PCI: percutaneous coronary intervention
