# Supplementary material for: Moderate-intensity versus high-intensity statin therapy in Korean patients with angina undergoing percutaneous coronary intervention with drug-eluting stents: A propensity-score matching analysis
Source: PLoS One. 2018 Dec 7;13(12):e0207889. doi: 10.1371/journal.pone.0207889 (PMC6286068; doi:10.1371/journal.pone.0207889)

**S1 Fig. Covariate balance in propensity-score matched patients between statin and non-statin groups.**


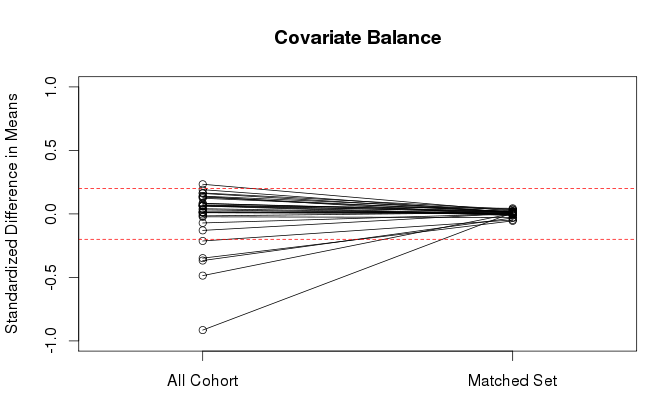

Supplement: S1 Fig — (DOCX) [file pone.0207889.s001.docx]
