# Supplementary material for: Moderate-intensity versus high-intensity statin therapy in Korean patients with angina undergoing percutaneous coronary intervention with drug-eluting stents: A propensity-score matching analysis
Source: PLoS One. 2018 Dec 7;13(12):e0207889. doi: 10.1371/journal.pone.0207889 (PMC6286068; doi:10.1371/journal.pone.0207889)
Supplement: S1 Table — (DOCX) [file pone.0207889.s003.docx]

**S1 Table. Specific anti-platelet agents in patients undergoing percutaneous coronary intervention with drug-eluting stents for angina according to statin therapy.**

|  | Overall (n=45,288) | | | Overall (n=32,936) | | | |
| --- | --- | --- | --- | --- | --- | --- | --- |
| Overall population | Statin  (n=39,509) | Non-statin  (n=5,779) | P Value | | Moderate-intensity statin  (n=23,863) | High-intensity statin  (n=9,073) | P Value |
| Medications at discharge, no. (%) |  |  |  | |  |  |  |
| Anti-platelet agents | 39,437 (99.8%) | 5,543 (95.9%) | <0.001 | | 23,821 (99.8%) | 9,055 (99.8%) | 0.666 |
| Aspirin | 38,000 (96.2%) | 5,020 (86.9%) | <0.001 | | 22,990 (96.3%) | 8,652 (95.4%) | <0.001 |
| Clopidogrel | 33,894 (85.8%) | 5,089 (88.1%) | <0.001 | | 21,348 (89.5%) | 7,068 (77.9%) | <0.001 |
| Prasugrel | 1,226 (3.1%) | 108 (1.9%) | <0.001 | | 653 (2.7%) | 355 (3.9%) | <0.001 |
| Ticagrelor | 4,157 (10.5%) | 230 (4.0%) | <0.001 | | 1,721 (7.2%) | 1,596 (17.6%) | <0.001 |
|  | Overall (n=11,166) | | | Overall (n=17,878) | | | |
| Matched population | Statin  (n=5,583) | Non-statin  (n=5,583) | P Value | | Moderate-intensity statin  (n=8,939) | High-intensity statin  (n=8,939) | P Value |
| Medications at discharge, no. (%) |  |  |  | |  |  |  |
| Anti-platelet agents | 5,511 (98.7%) | 5,517 (98.8%) | 0.099 | | 8,919 (99.8%) | 8,921 (99.8%) | 0.999 |
| Aspirin | 5,003 (89.6%) | 5,008 (89.7%) | 0.178 | | 8,565 (95.8%) | 8,536 (95.5%) | 0.824 |
| Clopidogrel | 4,999 (89.5%) | 5,074 (90.9%) | 0.537 | | 7,104 (79.5%) | 7,068 (79.1%) | 0.196 |
| Prasugrel | 107 (1.9%) | 108 (1.9%) | 0.783 | | 338 (3.8%) | 355 (4.0%) | 0.999 |
| Ticagrelor | 289 (5.2%) | 230 (4.1%) | 0.287 | | 1,443 (16.1%) | 1,462 (16.4%) | 0.289 |

Data are expressed as n (%).
