# Supplementary material for: Moderate-intensity versus high-intensity statin therapy in Korean patients with angina undergoing percutaneous coronary intervention with drug-eluting stents: A propensity-score matching analysis
Source: PLoS One. 2018 Dec 7;13(12):e0207889. doi: 10.1371/journal.pone.0207889 (PMC6286068; doi:10.1371/journal.pone.0207889)
Supplement: S2 Table — (DOCX) [file pone.0207889.s004.docx]

**S2 Table. Clinical outcomes according to statin therapy.**

| Clinical outcomes | Overall (n=45,288) | | |
| --- | --- | --- | --- |
|  | Statin  (n=39,509) | Non-statin  (n=5,779) | P Value* |
| All-cause death | 1,651 (4.2%) | 390 (6.7%) | <0.001 |
| Myocardial infarction | 313 (0.8%) | 34 (0.6%) | 0.046 |
| All-cause death/myocardial infarction | 1,918 (4.9%) | 410 (7.1%) | <0.001 |
| Clinical outcomes | Overall (n=32,936) | | |
|  | Moderate-intensity statin  (n=23,863) | High-intensity statin  (n=9,073) | P Value* |
| All-cause death | 1,050 (4.4%) | 317 (3.5%) | 0.172 |
| Myocardial infarction | 158 (0.7%) | 84 (0.9%) | 0.001 |
| All-cause death/myocardial infarction | 1,185 (5.0%) | 387 (4.3%) | 0.779 |

Values are presented as n (%). P-values were calculated using the log-rank test.*
