# Supplementary material for: Moderate-intensity versus high-intensity statin therapy in Korean patients with angina undergoing percutaneous coronary intervention with drug-eluting stents: A propensity-score matching analysis
Source: PLoS One. 2018 Dec 7;13(12):e0207889. doi: 10.1371/journal.pone.0207889 (PMC6286068; doi:10.1371/journal.pone.0207889)
Supplement: S3 Table — (DOCX) [file pone.0207889.s005.docx]

**S3 Table. Frequency and doses of statins in moderate- and high-intensity statin therapy groups.**

| **High-intensity statin (n=9,073)** |  |
| --- | --- |
| Atorvastatin 40 mg | 3,582 |
| Atorvastatin 80 mg | 1,411 |
| Rosuvastatin 20 mg | 4,080 |
| Rosuvastatin 40 mg | 0 |
| **Moderate-intensity statin (n=23,863)** |  |
| Atorvastatin 10 mg | 6,766 |
| Atorvastatin 20 mg | 6,701 |
| Rosuvastatin 5 mg | 293 |
| Rosuvastatin 10 mg | 7,532 |
| Simvastatin 20 mg | 525 |
| Simvastatin 40 mg | 44 |
| Simvastatin 80 mg | 0 |
| Pravastatin 40 mg | 453 |
| Pravastatin 80 mg | 0 |
| Lovastatin 40 mg | 0 |
| Pitavastatin 2 mg | 990 |
| Pitavastatin 4 mg | 439 |
| Fluvastatin XL 80 mg | 120 |
