# Supplementary material for: Moderate-intensity versus high-intensity statin therapy in Korean patients with angina undergoing percutaneous coronary intervention with drug-eluting stents: A propensity-score matching analysis
Source: PLoS One. 2018 Dec 7;13(12):e0207889. doi: 10.1371/journal.pone.0207889 (PMC6286068; doi:10.1371/journal.pone.0207889)
Supplement: S4 Table — (DOCX) [file pone.0207889.s006.docx]

**S4 Table. Characteristics of patients according to statin therapy.**

|  |  | Overall (n=39,227) | | | |
| --- | --- | --- | --- | --- | --- |
| Characteristics | Moderate-intensity statin  (n=23,863) | | High-intensity statin  (n=9,073) | 2≥statins or Ezetimibe containing statins  (n=6,291) | P Value |
| Age, years | 65.3±11.3 | | 63.4±11.7 | 64.3±11.6 | <0.001 |
| Gender male, no. (%) | 15,638 (65.5%) | | 6,337 (69.8%) | 4,199 (66.7%) | <0.001 |
| Enrolled number, no. (%) |  | |  |  | <0.001 |
| July 2009 to June 2010 | 5,665 (23.7%) | | 1,354 (14.9%) | 1,185 (18.8%) |  |
| July 2010 to June 2011 | 5,960 (25.0%) | | 1,714 (18.9%) | 1,537 (24.4%) |  |
| July 2011 to June 2012 | 6,363 (26.7%) | | 2,510 (27.7%) | 1,755 (27.9%) |  |
| July 2012 to June 2013 | 5,875 (24.6%) | | 3,495 (38.5%) | 1,814 (28.8%) |  |
| Comorbid conditions, no. (%) |  | |  |  |  |
| Diabetes | 7,724 (32.4%) | | 2,538 (28.0%) | 1,891 (30.1%) | <0.001 |
| Diabetes with chronic complications | 65 (0.3%) | | 31 (0.3%) | 19 (0.3%) | 0.559 |
| Hyperlipidemia | 10,798 (45.2%) | | 3,528 (38.9%) | 2,643 (42.0%) | <0.001 |
| Hypertension | 14,364 (60.2%) | | 4,843 (53.4%) | 3,431 (54.5%) | <0.001 |
| Congestive heart failure | 1,728 (7.2%) | | 484 (5.3%) | 405 (6.4%) | <0.001 |
| Arrhythmia | 2,038 (8.5%) | | 648 (7.1%) | 430 (6.8%) | <0.001 |
| Valvular disease | 117 (0.5%) | | 21 (0.2%) | 27 (0.4%) | 0.003 |
| Peripheral vascular disease | 2,921 (12.2%) | | 904 (10.0%) | 648 (10.3%) | <0.001 |
| Cerebrovascular disease | 3,366 (14.1%) | | 1,132 (12.5%) | 855 (13.6%) | 0.001 |
| Chronic pulmonary disease | 4,075 (17.1%) | | 1,403 (15.5%) | 1,015 (16.1%) | 0.001 |
| Moderate to severe liver disease | 16 (0.1%) | | 3 (0.03%) | 0 (0%) | 0.069 |
| Renal disease | 1,220 (5.1%) | | 321 (3.5%) | 306 (4.9%) | <0.001 |
| Cancer | 654 (2.7%) | | 224 (2.5$) | 141 (2.2%) | 0.058 |
| Rheumatic disease | 49 (0.2%) | | 20 (0.2%) | 10 (0.2%) | 0.707 |
| Charlson comorbidity index | 1.38±1.40 | | 1.17±1.28 | 1.28±1.35 | <0.001 |
| Number of drug-eluting stents | 1.41±0.65 | | 1.45±0.69 | 1.46±0.71 | <0.001 |
| Medications at discharge, no. (%) |  | |  |  |  |
| Anti-platelet agent | 23,821 (99.8%) | | 9,055 (99.8%) | 6,279 (99.8%) | 0.864 |
| Beta-blocker | 15,121 (63.4%) | | 6,511 (71.8%) | 4,426 (70.4%) | <0.001 |
| ACEI/ARB | 15,345 (64.3%) | | 5,837 (64.3%) | 4,393 (69.8%) | <0.001 |

Data are expressed as n (%) and mean ± SD.

ACEI = angiotensin converting enzyme inhibitor; ARB = angiotensin receptor blocker
